# Tuberculous Panophthalmitis with Lymphadenitis and Central Nervous System Tuberculoma

**DOI:** 10.1155/2016/6785382

**Published:** 2016-03-09

**Authors:** Sirawat Srichatrapimuk, Duangkamon Wattanatranon, Somnuek Sungkanuparph

**Affiliations:** ^1^Department of Medicine, Faculty of Medicine Ramathibodi Hospital, Mahidol University, Bangkok, Thailand; ^2^Department of Pathology, Faculty of Medicine Ramathibodi Hospital, Mahidol University, Bangkok, Thailand

## Abstract

Tuberculosis (TB) is a serious infectious disease that spreads globally. The ocular manifestations of TB are uncommon and diverse. TB panophthalmitis has been rarely reported. Here, we described a 38-year-old Thai man presenting with panophthalmitis of the right eye. Further investigation showed that he had concurrent TB lymphadenitis and central nervous system (CNS) tuberculoma, as well as HIV infection, with a CD4 cell count of 153 cells/mm^3^. Despite the initial response to antituberculous agents, the disease had subsequently progressed and enucleation was required. The pathological examination revealed acute suppurative granulomatous panophthalmitis with retinal detachment. Further staining demonstrated acid-fast bacilli in the tissue. Colonies of* Mycobacterium tuberculosis* were obtained from tissue culture. He was treated with antiretroviral agents for HIV infection and 12 months of antituberculous agents. Clinicians should be aware of the possibility of TB in the differential diagnosis of endophthalmitis and panophthalmitis, especially in regions where TB is endemic.

## 1. Introduction

Tuberculosis (TB) is still one of the leading causes of death worldwide, with an estimated annual incidence of 9 million and mortality of 1.5 million worldwide [1]. TB primarily affects the lungs but may also affect extrapulmonary organs. Remarkably, extrapulmonary manifestations of the disease are more common in immunocompromised individuals, including those with HIV infection [2]. Ocular TB is an uncommon extrapulmonary form of the disease. Any intraocular structure(s) may be involved in the infection, and myriad presentations have been reported [3–6]. TB endophthalmitis and panophthalmitis have been infrequently reported [7–25]. They represent an extreme end of the disease spectrum, with usually rapid progression of intraocular tissues destruction. In panophthalmitis, the sclera is also involved, which may result in globe perforation [3–6]. Here, we described an AIDS patient, who presented with TB panophthalmitis. He was also found to be afflicted with TB lymphadenitis and tuberculoma of the brain.

## 2. Case Presentation

A 38-year-old Thai man presented at the outpatient clinic with a chief complaint of painless blurred vision of the right eye for 3 weeks. He was a businessman at Samut Songkhram province and was previously healthy. He took no medicine, including herbal medicine. He had multiple sexual partners, although he claimed that he always used condom. He noticed the haziness of the whole picture, especially the central area, when he saw with his right eye. His conjunctiva was not injected. He recalled no history of prior eye trauma or surgery.

One week thereafter, the vision of right eye got worse. He also began to have right-sided headache. He then went to the local hospital where an unknown eye drop was prescribed, but no improvement of any symptoms was noted. Instead, his vision deteriorated, such that he had no light perception of the right eye. His right eyelid became swollen and right eye ptosis was noted. He began to experience right eye pain and more intense right-sided headache as well as intermittent fever. There was no history of cough, hemoptysis, weight loss, or any past history of TB or contact with a tuberculous patient. He decided to go to another hospital, where he was diagnosed with right orbital cellulitis. He was then referred to Ramathibodi Hospital.

**Figure 1:**
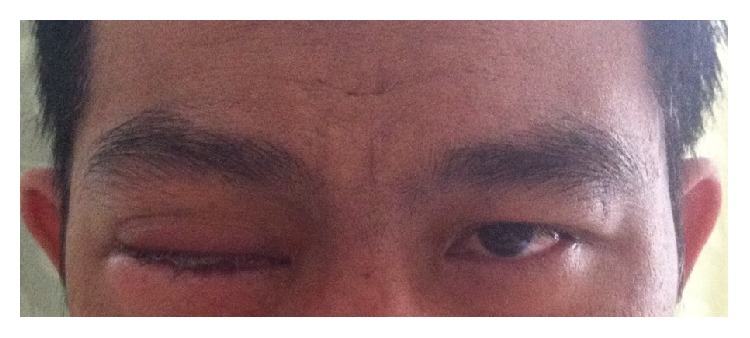
Patient's eyes at first presentation.

On examination, his temperature was 37.6°C. Other vital signs were within normal limits. He was a middle-aged male with sthenic built. His visual acuity was no perception of light (NPL) for right eye and 20/50 +2 with pinhole for left eye. Right eye ptosis and proptosis and chemosis were evident. Swelling, erythema, warmth, and tenderness on palpation of his right eyelid were also noted (Figure 1). His right cornea was opaque. His left eyelid, conjunctiva, and cornea were normal. The right pupil was 2 mm and nonreactive to light, while the left was 3 mm and reactive to light. Fundoscopy of the left eye revealed normal eye ground, whereas that of the right eye was not possible due to the opaque overlying cornea. Extraocular movement (EOM) of left eye was normal. By contrast, restricted EOM of the right eye was detected, with 20%, 10%, 10%, and 30% for upward, downward, lateral, and medial gaze, respectively. Ocular tensions of the right and left eyes were 32 and 16 mmHg, respectively. He had neither stiff neck nor any other neurological deficit. Oral thrush was detected. Examination of other systems was not revealing. Based on history and physical examination, the patient was diagnosed with endogenous endophthalmitis of right eye with suspected AIDS.

Blood tests were undertaken. His anti-HIV antibody was reactive, while HBsAg, anti-HCV antibody, and VDRL were negative. His CD4 cell count was 153 cells/mm^3^ (10%). His complete blood count and blood urea nitrogen as well as creatinine were all within reference ranges. His liver function test showed reverse AG ratio, with albumin level of 3.19 g/dL and globulin level of 5.72 g/dL, and mildly elevated ALT (85 U/L) and ALP (178 U/L). His chest film was normal. Blood culture for aerobic bacteria revealed no growth.

**Figure 2:**
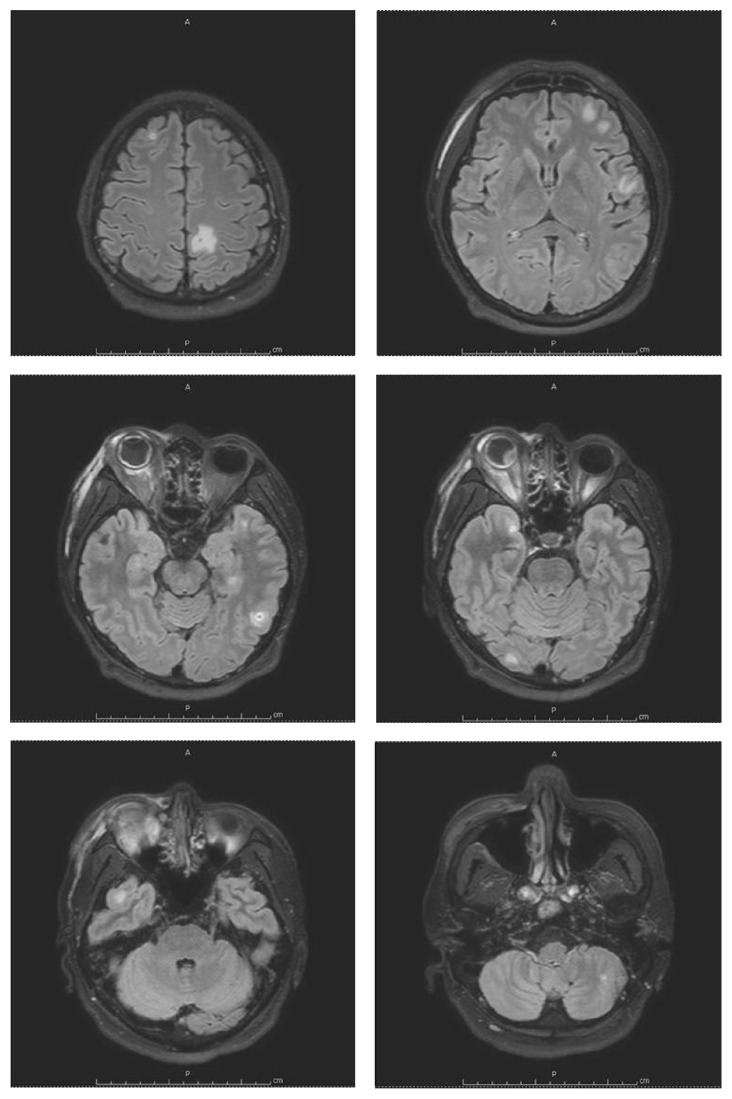
Brain MRI of the patient showing small ring-enhancing hypointense T1/hyperintense T2 lesion at inferomedial aspect in the right orbital globe, enhancement along retina and internal wall of the right orbital globe with suspected hyaloids detachment of the right eye, right exophthalmos, intra- and extraconal fat and periorbital soft tissue enhancement, perioptic neuritis of the right optic nerve without evidence of optic neuropathy, and multiple small round-shape enhancing lesions at gray-white junction of bilateral cerebrum and cerebellum. No leptomeningeal enhancement was detected.

Ultrasound of right globe revealed point-like lesion, loculated membrane-like lesion, and membrane-like lesion in the vitreous cavity. After-movements were seen on the dynamic scan. Brain and orbit MRI was then performed (Figure 2), which revealed small ring-enhancing hypointense T1/hyperintense T2 lesion at inferomedial aspect in the right orbital globe, enhancement along retina and internal wall of the right orbital globe with suspected hyaloids detachment of the right eye, right exophthalmos, intra- and extraconal fat and periorbital soft tissue enhancement, and perioptic neuritis of the right optic nerve without evidence of optic neuropathy. Altogether, these findings were suggestive of panophthalmitis of the right eye. In addition, there were multiple small round-shape enhancing lesions scattering at gray-white junction of bilateral cerebral hemispheres, periventricular white matter of the right parietal lobe, right caudate nucleus, left cerebellar hemisphere, left cerebellar tonsil, and right foramen of Luschka. Multiple subcentimeter lymph nodes were detected at bilateral parotid regions and bilateral levels IB and II with a necrotic lymph node at the deep lobe of the left parotid gland (not shown in the figure). Leptomeningeal enhancement was not found.

The patient was then subjected to additional investigations. Vitreous tapping of right eye was performed. Staining with Gram stain, modified AFB, AFB, and GMS was negative. No growth was detected from the culture for aerobic and anaerobic bacteria, fungus, and mycobacteria. PCR for detecting* M. tuberculosis* and* Toxoplasma gondii* were negative. PCR for 16S and 18S rRNA gene detection were also negative. Lumbar puncture was done, with clear colorless CSF obtained. Opening and closing pressure were 16 and 13 cm H_2_O, respectively. Cell count showed WBC 3 cells/mm^3^ (mononuclear 100%) without RBC. CSF total protein was 60 mg/dL. CSF sugar was 81 mg/dL (capillary blood glucose 106 mg/dL). Gram, modified AFB, and AFB staining revealed no organism. CSF cryptococcal antigen was negative. No growth was detected from the CSF culture for aerobic bacteria, fungus, and mycobacteria. PCR for detecting* M. tuberculosis*,* Toxoplasma gondii*, JC virus, and EBV of the CSF were all negative. No malignant cell was identified from CSF cytology.

Serum toxoplasma IgG and IgM were negative. PPD induration was 27 mm in diameter. Ultrasound of upper abdomen demonstrated diffuse hypoechoic change of the liver without focal lesion and prominent size of the spleen. No common bile duct or intrahepatic duct dilatation was evident. Gallstone was not found. Bedside transthoracic echocardiography showed no vegetation.

At the time, the differential diagnosis of TB or toxoplasmosis was entertained. Intravenous and intravitreous vancomycin plus ceftazidime were empirically given during the investigations. Systemic antibiotics were continued for 12 days. Thereafter, the antibiotics were changed to oral cotrimoxazole (12 mg/kg/day of trimethoprim) and antituberculous drugs (isoniazid/rifampicin/pyrazinamide/ethambutol/vitamin B6). Five days after treatment change, bilateral cervical lymph node enlargement was observed. Thus, FNA was carried out by the ENT consultant. Two milliliters of bloody content was obtained. While staining with Gram stain, modified AFB, AFB, and GMS was not revealing, PCR for detecting* M. tuberculosis* was positive. Mycobacterial culture of the content also grew pan-sensitive* M. tuberculosis* complex, whereas aerobic and fungal culture grew no organism. Cytology showed necrotic cellular debris, degenerative leucocytes, and monocytes, consistent with necrotizing inflammation. As a result, the patient was diagnosed with AIDS with TB panophthalmitis, TB lymphadenitis, and suspected CNS tuberculoma, and antituberculous drugs were continued. However, his treatment was complicated by drug-induced hepatitis. Antituberculous regimen was hence adjusted by having ofloxacin in place of pyrazinamide. The patient demonstrated some response to treatment, with less pain and swollen eye, improvement of right EOM, and defervescence, although the visual acuity remained the same.

Follow-up brain and orbit MRI, performed 2 weeks after receiving antituberculous drugs and cotrimoxazole, showed decreased degree of enhancement of the globe, the optic nerve, the intra- and extraconal fat, and the periorbital soft tissue of the right orbit, as well as decreased number, size, and degree of enhancement of multiple scattered small enhancing nodules at the cerebrum and cerebellum. Perilesional vasogenic edema also decreased. The patient was discharged home with antituberculous drugs and cotrimoxazole. He insisted on good drug compliance.

**Figure 3:**
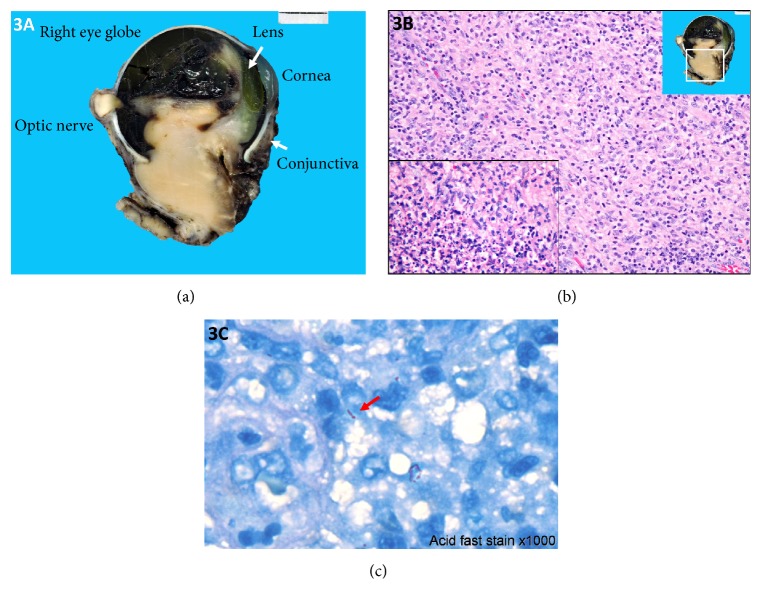
(a) Gross specimen of the enucleated right eye showing mass-forming lesion involving vitreous chamber, sclera, uveal tissue, conjunctiva, and periorbital soft tissue. (b) Histopathological findings of the specimen showing mixed inflammatory cell infiltration composed of lymphocytes, plasma cells, histiocytes, and some area of suppurative granulomatous inflammation (lower inset). (c) Acid-fast staining demonstrated acid-fast bacilli in the tissue (arrow).

However, around 4 weeks thereafter, his right eye became, again, swollen and tender. There was little yellow discharge from the eye. On examination, marked chemosis with yellow discharge as well as localized abscess at subconjunctival space was present. No cervical lymphadenopathy was detected. Right eye enucleation was then undertaken. Gross examination showed mass-forming lesion involving vitreous chamber, sclera, uveal tissue, conjunctiva, and periorbital soft tissue (Figure 3(a)). The pathological examination revealed acute suppurative granulomatous panophthalmitis with retinal detachment (Figure 3(b)). Further staining of the tissue demonstrated acid-fast bacilli in the tissue (Figure 3(c)). GMS, PAS, Brown-Brenn, and Warthin-Starry staining was negative. Mycobacterium culture of the tissue grew pan-sensitive* M. tuberculosis* complex, whereas aerobic and anaerobic bacterial as well as fungal culture grew no organism.

The patient was finally diagnosed with AIDS with TB panophthalmitis, TB lymphadenitis, and CNS tuberculoma. He was treated with 2 months of isoniazid/rifampicin/ethambutol/ofloxacin, followed by 1 month of isoniazid/rifampicin/ethambutol/levofloxacin and 9 months of isoniazid/rifampicin. Cotrimoxazole was continued for 6 weeks.

Because of the problems of antituberculous drug-induced hepatitis and the progression of eye symptoms that mandated right eye enucleation, antiretroviral therapy could not be initiated in the first few weeks. Antiretroviral agents (tenofovir/lamivudine/efavirenz) were commenced 6 weeks after antituberculous drugs initiation. The patient had a regular follow-up and good adherence to the regimen. His HIV viral load was undetectable (<40 copies/mL), whereas his CD4 count rose to 288 cells/mm^3^ (17%), after 6 months of antiretroviral therapy.

## 3. Discussion

Ocular TB is a rare manifestation of extrapulmonary TB. Due to the low sensitivity of confirmatory laboratory investigation and inconsistency of diagnostic criteria across different studies, there are no reliable data of its prevalence. Ocular involvement in patients with systemic TB was reported to be 1.46% in the study from US [26] and 18% in the study from Spain [27]. According to several reports from various countries, different proportions of patients with uveitis were ascribed to TB, from 0.5% to 10.5% [28–32].

The disease occurs after hematogenous seeding of the bacilli, or, less commonly, by extension of infected nearby tissues [3–6]. Furthermore, the immune response to the bacilli can cause a hypersensitivity reaction in different parts of the eye [3–6]. The onset of disease is usually insidious. Clinical spectrum of the disease may be nonspecific and may vary according to the involved structure, ranging from uveitis (most common), retinitis, retinal vasculitis, optic neuropathy, keratitis, and keratoconjunctivitis to endophthalmitis and panophthalmitis [3–6].

TB endophthalmitis and panophthalmitis have been infrequently reported (Table 1). From the literature, most patients were immunocompetent, even though comprehensive analyses of immune status were not performed and HIV testing was not done in the early studies. In those who were infected with HIV, CD4 cell counts were low (range: 34–263 cells/mm^3^) [23]. The onset of the disease is usually insidious, in the range of months. The disease in nearly all patients is unilateral. Most patients also had TB involvement of other sites, which were either concurrently or subsequently detected. This is in contrast to ocular TB with different manifestations, for example, choroiditis, which are more commonly reported to occur in isolation [3–6, 21]. The prognosis is usually grave, with enucleation/evisceration required in nearly all cases. Without such procedures, successful treatment of actual TB endophthalmitis case was described in only 2 reports [24, 25]; prompt vitrectomy along with the administration of antituberculous agents might be the key success factor. Pathology of the tissue usually shows granulomatous inflammation and the presence of acid-fast bacilli.

Given the protean manifestations of ocular TB and usually insensitive laboratory investigations, delayed diagnosis and misdiagnosis are not uncommon. These may, however, lead to a devastating consequence. For example, patients initially presenting with ocular symptoms resembling ocular malignancy or other inflammatory conditions may have been treated incorrectly. Steroid was given to the patients with uveitis of unknown etiology, which later evolved into TB endophthalmitis [9, 10, 13, 21]. Thus, clinicians should keep in mind the possibility of TB in the differential diagnosis of uveitis, retinitis, retinal vasculitis, endophthalmitis, and panophthalmitis, especially in the presence of extraocular focus of TB and/or the absence of other etiologies. Clinical specimen should also be sent for AFB staining, mycobacterium culture, and PCR for mycobacterium detection. In addition, searching for evidence of other organ involvements, for example, chest film, sputum AFB staining, and PPD skin test, might provide clue for presumptive diagnosis of ocular TB [4]. In certain situations, therapeutic trial with antituberculous agents (4-drug regimen for 4–6 weeks) may be necessary [4–6, 33]. Guidelines for diagnosis of intraocular TB have been proposed [4]. In ocular TB, standard treatment regimens, as in other extrapulmonary or pulmonary TB treatment, are recommended [4, 5, 33, 34]. The patients usually achieve good response [4, 5, 33, 34].

Extrapulmonary TB is more common in patients coinfected with HIV. Babu and colleagues examined HIV patients with ophthalmic complaints and/or with CD4 count below 200 cells/mm^3^ and found that only 1.95% of such patients were affected by ocular TB [23]. Recently, Sudharshan and colleagues reported ocular findings of 1,000 HIV patients visiting a tertiary eye care center of India; ocular TB was found in 3.8% of the patients [35]. Similar to pulmonary TB, ocular TB occurred over a wide range of CD4 count, although it was more common in patients with CD4 count below 300 cells/mm^3^ [23, 35]. HIV-infected patients with TB endophthalmitis or panophthalmitis were reported in only a few cases [23, 35] and manifested similar to those in non-HIV-infected patients.

In our case, the presentation was similar to those reported previously (Table 1). The patient had no evidence of pulmonary TB but had concurrent TB cervical lymphadenitis and suspected CNS tuberculoma. Even though we did not get the tissue from the CNS lesion for establishing the diagnosis, all of the organs (eye, lymph node, and brain) were most likely affected by the same organism. Of note, multiple ring-enhancing lesions in the brain in patients with TB panophthalmitis have also been described elsewhere [18, 19, 35]. These lesions showed improvement after antituberculous agent administration [18, 19].

Interestingly, the patient showed worsening of the symptoms after an initial response, albeit he had good drug compliance and the bacilli were susceptible to the drugs. It is possible that this represents a “paradoxical reaction” to the treatment [36]. Given the fact that antiretroviral agents were initiated only 1 week prior to the deterioration, paradoxical reaction, rather than immune reconstitution inflammatory syndrome (IRIS), is more likely responsible for the event. We did not have the data of patient's CD4 count at the time of the enucleation. Notably, a recent report described similar reexacerbation of inflammation after 2 months of TB endophthalmitis treatment with isoniazid, rifampicin, and ethambutol in a non-HIV-infected patient [25]. The symptoms were not improved by adding systemic antibacterial and antifungal drugs but abated by a tapering regimen of oral prednisolone [25].

In summary, we described an AIDS patient with TB panophthalmitis, TB lymphadenitis, and CNS tuberculoma. TB endophthalmitis and panophthalmitis are sight-threatening and usually have insidious onsets. Most cases demonstrated systemic TB involvement. In HIV-infected patients, ocular TB occurred over a wide range of CD4 count. Until better diagnostic modalities are available, a high index of clinical suspicion and early treatment are vital for the ocular TB treatment outcome.

## Figures and Tables

**Table 1:** Clinical features of TB endophthalmitis/panophthalmitis cases previously described in the literature.

Authors	Year	Number of cases	Host (sex/age/immune status)	Onset	Extraocular foci of TB	Outcomes	Notes
Dvorak-Theobald [7]	1958	1	M/37/immunocompetent	2 weeks	TB adrenal gland, previous TB kidney	Enucleation	—

Arrell [8]	1967	1	M/73/immunocompetent	2 months	Old pulmonary TB (from chest film)	Evisceration	—

McMoli et al. [9]	1978	1	M/1/immunocompetent	?	Pulmonary TB	Evisceration	—

Manthey et al. [10]	1982	1	F/60/immunocompetent	?	Pulmonary TB	Enucleation	Prior steroid treatment led to temporary regression

			F/30/immunocompetent	2 months	Previous disseminated TB, active pulmonary TB	Enucleation	—
Ni et al. [11]	1982	3	M/11/immunocompetent	2 months	Abnormal chest film	Enucleation	Misdiagnosed with retinoblastoma
			F/15/immunocompetent	1 month	Abnormal chest film	Enucleation	—

Menezo et al. [12]	1987	1	F/20/HIV	3 days	None but PPD+	Evisceration	History of heroin use

Regillo et al. [13]	1991	1	F/29/immunocompetent	6 months	Pulmonary TB	Evisceration	Prior steroid treatment

Anders and Wollensak [14]	1995	1	F/36/SLE	2 months	Pulmonary TB	Enucleation	—

Biswas et al. [15]	1995	2	F/42/?	?	TB liver	Evisceration	—
			F/30/?	?	Pulmonary TB	Evisceration	Bilateral involvement

Raina et al. [16]	2000	1	F/8/immunocompetent	3 months	None but PPD+	Enucleation	—

Sheu et al. [17]	2001	2	F/75/immunocompetent	2 weeks	Pulmonary TB	Evisceration	—
			M/68/immunocompetent	2 months	Pulmonary TB (miliary TB)	Enucleation	—

Grosse et al. [18]	2002	1	M/30/immunocompetent	2 months	TB lymphadenitis (generalized), CNS tuberculoma	Evisceration	—

Sen et al. [19]	2003	1	M/4/immunocompetent	1 month	CNS tuberculoma	Evisceration	Bilateral involvement

Chawla et al. [20]	2004	1	F/12/immunocompetent	2 months	Abnormal chest film responsive to ATT	Enucleation	—

Demirci et al. [21]	2004	2	M/28/immunocompetent	3 months	TB peritonitis (later)	Evisceration	Prior steroid treatment
			F/29/immunocompetent	5 months	Granulomatous hilar lymphadenopathy, PPD+	Enucleation	Prior steroid treatment

			M/45/HIV, CD4 = 88	?	Pulmonary TB	Evisceration	—
Babu et al. [23]	2006	3	M/36/HIV, CD4 = 263	?	Pulmonary TB	Evisceration	—
			M/34/HIV, CD4 = 34	?	Pulmonary TB, abdominal TB	Evisceration	—

Mehta and Vaidya [24]	2011	1	F/77/immunocompetent	1 week	None, PPD and QFT—negative, CT chest and abdomen—normal	Regressed with ATT/vitrectomy	Underwent cataract extraction 2 months earlier

Hase et al. [25]	2015	1	M/81/steroid use	?	Pulmonary TB (miliary TB)	Regressed with ATT/vitrectomy	—

TB = tuberculosis, M = male, F = female, SLE = systemic lupus erythematosus, PPD = purified protein derivative (Mantoux) test, QFT = Quantiferon TB gold test, and ATT = antituberculous therapy.
